# The effect of changing the moisture levels of dry extruded and wet canned diets on physical activity in cats

**DOI:** 10.1017/jns.2017.9

**Published:** 2017-04-17

**Authors:** D. G. Thomas, M. Post, G. Bosch

**Affiliations:** 1 Centre of Feline Nutrition, Institute of Veterinary, Animal & Biomedical Sciences, Massey University, Private Bag 11 222, Palmerston North, New Zealand; 2 Animal Nutrition Group, Department of Animal Sciences, Wageningen University, Wageningen, The Netherlands

**Keywords:** Cats, Physical activity, Dietary moisture, Total water intake, Urine production, BW, body weight, FAA, food anticipatory activity, USG, urine specific gravity

## Abstract

Obesity levels in cats are increasing and the main causative factor is higher energy intake *v*. energy expenditure over time. Therefore, altering energy expenditure by enhancing physical activity of the cat could be a strategy to reduce obesity. Hydrating commercial dry diets with water increased activity in cats; however, no study has compared this approach with feeding high-moisture canned diets. Eight healthy male neutered domestic shorthair cats were fed four different dietary treatments in a Latin square design. Treatments were a canned diet ‘as is’ (82 % moisture) and freeze-dried (4 %), a dry diet ‘as is’ (3 %) and with added water (70 %). Cat activity was measured continuously using Actical^®^ accelerometers. Cats were group housed during the first 14 d of each period and then moved to individual cages for 7 d with faecal and urine production measured over the final 4 d. Intake was similar for each diet. The average activity over 24 h was not different between treatments (*P* > 0·05). However, the ratio between average activity during the day *v*. at night was higher when cats were fed the dry diet (*P* = 0·030). Total water intake and urine volume increased when the canned diet was fed (*P* < 0·001). The similarity in total activity of the cats on the treatments indicates that dietary moisture or diet type did not have a major effect on these cats. However, the stronger diurnal activity patterns observed in the cats when they were fed the dry diet are intriguing and require further study.

Obesity is now recognised as the most important medical disease in pets worldwide^(^^1^^)^. Recent studies continue to report very high incidences of overweight and obese animals in different pet cat populations around the world^(^^2^^,^^3^^)^. However, despite extensive research activity and owner education initiatives obesity levels remain stubbornly high, with the main causative factor being higher energy intake *v*. energy expenditure over time. Current strategies for weight management involve dietary restriction with purpose-formulated light or weight-control diets^(^^1^^)^, rather than strategies to increase energy expenditure via increased activity. However, recent research where commercial dry diets have been hydrated resulted in increased activity levels in cats housed indoors^(^^4^^–^^6^^)^. While no study to date has compared this approach with feeding high-moisture canned diets, other work has shown that the water content of canned diets induces decreased voluntary energy intake and body weight (BW) in cats fed *ad libitum*^(^^7^^)^. The present study aimed to investigate the effect of hydration of a dry extruded diet and dehydration of a wet canned diet on activity in a group of cats.

## Materials and methods

The study reported here was approved by the Animal Ethics Committee, Massey University, Palmerston North, New Zealand (protocol 15/45). Eight healthy adult male neutered domestic shorthair cats were used in the study with an average age of 2·5 (sd 0·33) years, an average BW of 4·19 (sd 0·20) kg and an average body condition score of 5·19 (sd 0·23) on a nine-point scale^(^^8^^)^. BW was measured twice-weekly and body condition score assessed weekly during the study.

Four different dietary treatments were fed in a Latin square design over four 21-d periods. Two treatments were based on a commercial canned diet (Chef Chicken and Rabbit; Heinz Wattie's Ltd; containing 56·0 % crude protein, 29·3 % crude fat and 8·3 % nitrogen-free extract on a DM basis) and two on a commercial dry diet (Royal Canin Adult Fit 32; Mars Inc.; containing 33·3 % crude protein, 15·2 % crude fat and 38·3 % nitrogen-free extract on a DM basis). Specifically the four treatments were the canned diet fed ‘as is’ (WW: 82 % moisture) and freeze-dried (WD: 4 % moisture), and the dry diet fed ‘as is’ (DD: 3 % moisture) and with added water (DW: 70 % moisture). Water was added to the daily allowance of dry food, which was stored in a closed box before feeding using the method of Alexander *et al.*^(^^6^^)^.

Cats were fed 50 % of their daily allowance in food bowls at 09.00 hours and the other 50 % at 15.00 hours with 1 h to consume their food allocation. Their daily allowance was calculated based on energy requirements (418 kJ/kg BW^0·67^)^(^^9^^)^. Food intake was measured after each meal throughout the trial by accurately weighing each food bowl. Cats were group housed during the first 14 d of each period in two groups of four animals, and were housed in individual cages for the last 7 d with faecal and urine production monitored for the final 4 d. Each morning during the last 7 d of each period, the cats were provided with accurately weighed bowls of fresh water. Water bowls from the previous day were accurately reweighed to allow calculation of daily water intake.

Activity was measured continuously during each 21 d period using Actical^®^ accelerometers (Mini Mitter)^(^^10^^,^^11^^)^. The Actical^®^ devices were attached to each cat's collar and removed for 1 h every week to allow downloading of activity data to the computer. Activity was measured as activity counts per epoch (15 s) and these were combined to give activity counts per 24 h, during daylight and night-time hours. Anticipatory activity before feeding (2 h before each feeding time as a percentage of total daily activity; food anticipatory activity (FAA)) was also calculated.

During the 4-d collection period faecal and urine output was measured and recorded daily. Each day the litter trays were removed between 08.00 and 09.00 hours. A litter tray containing a mesh insert, without litter, was used to separate faeces and urine. Total urine and faecal output was measured and faeces were scored on a scale from 1 to 5, where 1 is hard dry faeces and 5 is watery diarrhoea^(^^12^^)^. Faecal samples were weighed and then stored in a freezer at −20°C until analysis of water content. Urine specific gravity (USG) was determined using an American Optical refractometer (Atago Co. Ltd).

For determination of faecal moisture, the frozen faecal samples were freeze-dried (Cuddon Engineering) and ground to pass a 1-mm screen using an ultra-centrifugal mill (model ZM100; Retsch GmbH and Co.). Faecal moisture content was then determined by drying the faeces to a constant weight at 103°C and calculating the total weight loss of the sample.

### Statistical analyses

Data were analysed separately for effect of treatment diet on activity counts, urine and faecal output, faecal moisture and USG. Treatment effects were tested for significance (*P* < 0·05) using the PROC MIXED procedure in SAS (SAS version 9.3; SAS Institute, Inc.). The statistical model used for analyses of activity data was:

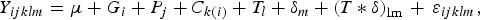

where *Y*_*ijklm*_ = treatment effect, *μ* = mean, *G*_*i*_ = effect of group (pen), *P*_*j*_ = effect of period, *C*_*k*(*i*)_ = effect of cat within pen, *T*_*l*_ = effect of treatment (diet), *δ*_*m*_ = effect of period, (T*δ)_lm_ = interaction effect of treatment and time and *ε*_*ijklm*_ = residual error.

For the analyses of urine and faecal output, faecal moisture and USG, the effect of time and the interaction effect of treatment and time were excluded from the model (*P* > 0·05). Moreover, effect of group was excluded from the model for the analyses of urine and faecal output, faecal moisture and USG, and effect of cat within pen became effect of cat, because cats were single-housed during these measurements. Least square means and the pooled standard errors were reported for all response criteria.

## Results

All cats remained healthy during the experiment and diets were generally well accepted. The average intake of each diet was similar (WW: 1505 (sem 62·9) kJ/d, WD: 1518 (sem 64·8) kJ/d, DD: 1402 (sem 70·5) kJ/d, DW: 1312 (sem 64·9) kJ/d; *P* = 0·262). Average BW values of cats were different between treatments (WW: 3945 (sem 68·4) g, WD: 3990 (sem 80·3) g, DD: 3903 (sem 75·1) g, DW: 3924 (sem 78·3) g; *P* < 0·001) and period (*P* < 0·001). The body condition score of cats remained constant during the experiment (*P* > 0·05 for period).

The average activity over 23·9 h, average activity during the light (day) and dark (night) period and FAA were not different between treatments (*P* > 0·05; Table 1). However, the ratio of the average activity counts during the light period *v*. the dark period was higher when cats were fed the high-carbohydrate dry diet, compared with all other treatments (*P* = 0·030). Approximately 2 h before each feeding time activity increased. This FAA accounted for 33 % of the total daily activity (see Table 1), observed for each cat and was similar for all treatments (*P* = 0·938).

**Table 1.** Effect of the four dietary treatments on activity in cats (Mean values and pooled standard errors)

	Activity (counts/epoch of 15 s)				
	Wet diet	Freeze-dried wet diet	Dry diet	Dry diet with added water	sem
Total	111·0	110·0	122·0	124·5	155·4
Light	160·0	165·5	172·8	180·2	264·3
Dark	60·0	61·4	59·1	62·6	112·9
Light:dark ratio	3·82^b^	3·61^b^	5·64^a^	4·79^b^	1·31
FAA, total (%)*	32·8	33·3	33·8	33·7	5·4
FAA, morning (%)*	20·5	20·0	18·8	17·7	4·3
FAA, afternoon (%)*	14·4	14·3	14·6	15·4	2·4

FAA, food anticipatory activity.

^a,b^ Mean values within a row with unlike superscript letters were significantly different (*P* < 0·05; *n* 8 cats per treatment).

* FAA calculated as activity 2 h before each feeding time as a percentage of total daily activity.

Total water intake increased when the wet diet was fed, compared with all other treatments (*P* < 0·001; Table 2). Water intake by drinking was lowest for cats on the wet diet and cats on the dry diet with added water. Dietary water intake was highest when the wet diet was fed, intermediate for the dry diet with added water and lowest when the dry diet and the freeze-dried wet diet were fed (*P* < 0·001).

**Table 2.** Average daily water intake, faecal and urine production and urine specific gravity of cats on the four different dietary treatments (Mean values and pooled standard errors)

	Diets				
	Wet	Dry	Freeze-dried wet	Dry with added water	sem
Drinking water intake (ml)	0·7^c^	101·1^b^	161·5^a^	3·7^c^	20·6
Dietary water intake (ml)	286·0^a^	13·8^c^	3·9^c^	150·2^b^	17·4
Total water intake (ml)	293·0^a^	109·2^c^	157·9^b^	153·5^b^	16·6
Faecal production (g)	50·2	51·3	59·3	62·3	11·5
Faecal water (%)	72·6	68·6	66·7	71·0	3·3
Faecal score*	2·8	2·9	2·6	3·3	0·5
Urine volume (ml)	228·2^a^	61·5^c^	101·8^b^	92·7^b^	16·9
Urine specific gravity	1·028^c^	1·064^a^	1·059^a^	1·043^b^	0·0
Water balance (ml)^†^	28·4	12·5	16·5	16·6	–

^a,b,c^ Mean values within a row with unlike superscript letters were significantly different (*P* < 0·05; *n* 8 cats per treatment).

* On a scale from 1 to 5, where 1 is hard dry faeces and 5 is watery diarrhoea^(^^12^^)^.

† Calculated as total water intake – faecal water output – urine volume.

Faecal production, faecal water and faecal score were similar for all dietary treatments (*P* > 0·05). Urine volume was highest when cats were fed the wet diet and was lowest for cats on the dry diet (*P* < 0·001). In accordance with the urine volume, the USG was lowest for cats on the wet diet and highest for cats on the dry diet and the freeze-dried wet diet (*P* < 0·001).

## Discussion

Consumption of dry diets with added water has previously been observed to increase physical activity in cats^(^^4^^–^^6^^)^. However, the mechanisms behind this increase in activity are unclear. If activity is simply driven by hydration status, a higher activity for the cats on the high-moisture diet and cats on the dry diet with added water in the present study would have been expected, but this was not apparent.

Other factors which may affect activity are hunger and satiety. Cats may be more active before feeding when they are hungry, which has been hypothesised as instinctive food-searching behaviour^(^^4^^)^. In the study of Cameron *et al*.^(^^4^^)^ cats fed on a restricted diet (80 % of their individual habitual energy intake) had higher total activity and activity peaks before morning feeding when compared with cats fed *ad libitum*. If activity is influenced by hunger, it would be hypothesised that higher-satiating diets would result in lower FAA as found in dogs^(^^13^^)^. However, FAA before the morning or afternoon feeding in the present study was similar between treatments, suggesting there were no differences in the satiety levels of the cats in this preprandial period. In the study of Deng *et al*.^(^^5^^)^, 2 h FAA before the afternoon meal was lower (*P* = 0·049) in cats fed a 70 % hydrated dry diet than in cats fed a non-hydrated dry diet (the same two diets as were used in the present study). FAA before the morning meal and total FAA were, however, not different among treatments. The total FAA as a percentage of total activity in the study of Deng *et al*.^(^^5^^)^ was slightly higher than FAA in the present study (37 *v*. 33 %). However, there were large differences in total activity between the present study (116·87 activity counts/epoch) and the previous work^(^^5^^)^ (29·80 activity counts/epoch). Variation in activity counts between animals in the present study was higher than in previous work, which was mainly caused by high activity counts in two of the eight cats. No behavioural observations were carried out during this study, so it is unknown what specific behaviours were causing this difference. It is possible that the inter-animal variation in activity counts could have been caused by differences in sensitivity between the accelerometers, making it difficult to compare total activity results between the present study and previous work. However, each cat in the present study received each diet for the same amount of time and the activity monitors were not switched between cats during the experiment so the relative effects of dietary treatment within the present study can still be assessed.

The feeding times, diets and number of cats used were similar between the two studies, but cats were housed inside in the Deng *et al*.^(^^5^^)^ study compared with outside in the present study which might have affected cat behaviour. Deng *et al*.^(^^5^^)^ also reported a lower light:dark ratio of activity in cats fed the dry diet (2·84) and hydrated dry diet (2·19) which compared with 5·64 and 4·79 for the same two treatments in the present study. A reason for this difference may be that cats in the previous study were housed inside, with an 16:8 h light–dark cycle^(^^5^^)^, while cats in the present study were housed outside, under a natural light–dark cycle which averaged 9·5:15·4 h. Activity was compared by calculating the activity counts/epoch, so the longer light period in the previous study^(^^5^^)^ might explain the differences in ratios compared with the present study. However, despite these differences, the ratio between average activity during the day *v*. at night was significantly higher when cats were fed the dry diet, which agrees with Deng *et al*.^(^^5^^)^ and requires further study.

An interesting finding in the present study was the difference in the total water intake between the different dietary treatments. The significantly higher water intake of cats on the wet diet agrees with previous research^(^^14^^,^^15^^)^, and indicates that cats on a low-dietary moisture diet do not fully compensate by drinking water to reach the same total water intake as when they were a fed high-moisture diet.

Water requirements of cats are determined using a 1:1 ratio between water and metabolic energy intake^(^^9^^)^. For the present study, this equates to an average total daily water intake of 253 ml which was only reached when the wet diet was fed. However, when comparing the drinking water intake component of the present study with previous work, water intake when feeding the high-moisture diets was lower^(^^16^^)^. In the study of Buckley *et al*.^(^^16^^)^ a 73·7 % moisture diet was fed and drinking water intake was 30 ml/kg BW per d; for the present study this was 0·17 ml/kg BW per d when the wet diet was fed and 0·88 ml/kg BW per d when the dry with added water diet was fed. It appears that the higher moisture content of the canned diet in the present study (82 %) provided the great majority of the cats’ requirements. Drinking water intake when the dry diet (24·14 ml/kg BW per d) was close to that in the study of Buckley *et al*.^(^^16^^)^ where a dry diet with a 6·3 % moisture content was fed (21·1 ml/kg BW per d). However, the drinking water intake for the freeze-dried wet diet (38·57 ml/kg BW per d) was substantially higher. This difference in drinking water intake between the dry diet and the freeze-dried wet diet is interesting. Considering the comparable moisture content of these diet (3 and 4 %), a similar total water intake would be expected. The difference between the dietary treatments might be a result of the difference in food structure. The brittle, dry structure of the freeze-dried wet diet might have made the ingestion of the diet harder, which might have motivated cats to drink more than when fed the dry diet. Furthermore, macronutrient content of the diets and the stickiness due to gelling agents might have had an influence on postprandial water intake. Other species increase their water intake when dietary protein levels are higher^(^^17^^)^.

Increased water intake is related to relative supersaturation of calcium oxalate and a reduction of USG. In the study of Buckley *et al*.^(^^16^^)^ a dry diet was hydrated with water to obtain four dietary treatments with moisture levels of 6·3, 25·4, 53·2 and 73·3 %. Water intake increased when dietary moisture level increased. With a total water intake of 144·7 ml/d for the 73·3 % moisture diet compared with 103·4, 98·6 and 104·7 ml/d for the 53·2, 25·4 and 6·3 % moisture diets, respectively. USG was reduced after feeding the high-moisture diet (1·036) compared with the lower-moisture diets (1·052–1·054). This reduction is also observed in the present study, i.e. 1·028 for the wet diet (82 % moisture) compared with 1·059–1·064 for the other diets.

In conclusion, the lack of difference in total activity of the cats on the dietary treatments seems to indicate that dietary moisture did not have a major effect in cats kept outdoors. However, the stronger diurnal activity patterns observed in the cats when they were fed the dry diet *v*. all other treatments are intriguing and require further study.
